# A narrative review and scoring proposal for secondary lumbar instability after lumbar decompression surgery

**DOI:** 10.1007/s00701-025-06590-9

**Published:** 2025-06-18

**Authors:** Anto Abramovic, Sara Lener, Sebastian Hartmann, Claudius Thomé

**Affiliations:** Department of Neurosurgery, Medical University of Innsbruck, Anichstrasse 35, Innsbruck, A-6020 Austria

**Keywords:** Secondary instability, Postoperative olisthesis, Lumbar decompressive surgery, Postoperative slippage

## Abstract

**Purpose:**

Lumbar spinal stenosis (LSS) is a common condition in the aging population, where decompressive surgery (DS) is widely regarded as the gold standard due to its effectiveness in relieving symptoms. However, DS carries the risk of secondary lumbar instability (SLI), while fusion surgery, although mitigating this risk, may lead to overtreatment and complications such as adjacent segment disease. The aim of the present study was to review the current literature on preoperative radiological and clinical variables, thus accounting for SLI after lumbar decompression surgery and to derive a score for SLI risk prediction.

**Methods:**

A literature review using online databases was performed in order to identify risk factors for the emergence of SLI. Risk factors were then graded for relevance. Consequently, a risk score for predicting SLI was developed from these results.

**Results:**

25 studies including 9754 patients were identified. The most commonly described predictors for SLI were preoperative instability, disc height > 6.5 mm, surgical invasiveness as well as patient-related risk factors such as BMI, age, gender and presence of mechanical low back pain. Based on these results, a 14-point scale was created using the most relevant risk factors selected by the research group using a peer-review process.

**Conclusion:**

The proposed score identifies known risk factors for SLI, rated according to their importance on clinical decision making. This represents an initial theoretical approach that has to be validated by prospective clinical studies. Nevertheless, decision making may already be supported by the awareness of the characterized risk factors.

## Introduction

Lumbar spinal stenosis (LSS) is a common disease in the aging population with a growing socioeconomic importance [2, 60]. Typical symptoms include neurogenic claudication, low back pain (LBP) and radiating pain, characteristically increasing when standing and walking, and showing significant impact on mobility and quality of the patient’s life. Treatment options include conservative management, as well as surgery, primarily aiming at decompression of neural structures. Previous research clearly demonstrated the superiority of surgical over conservative treatment [3, 4, 16, 26, 56, 70–72]. Decompression surgery of LSS has been considered the “gold standard” [58]. The traditional laminectomy technique allows an extensive decompression of neural structures, but carries well reported disadvantages due to extensive muscle trauma and the risk for secondary lumbar instability (SLI), contributing to a poor outcome in up to 50% of patients [21, 52, 68, 75]. Some landmark studies recommended additional fusion and since then the annual rate of fusion surgeries has increased significantly [6, 11, 17, 35]. To overcome the outlined disadvantages of laminectomy, less invasive microsurgical decompression techniques have been developed [18, 68]. These techniques, however, also differ and have a varying impact on segmental stability [36, 42]. Reoperation rates and the likelihood of secondary fusion procedures cannot be neglected, especially in patients with degenerative spondylolisthesis [5]. Nevertheless, management protocols for LSS and accompanying degenerative fixed or low-grade spondylolisthesis are still controversial, and standard guidelines favoring a decompression or fusion procedure are lacking. While lumbar decompression surgery (LDS) as a"stand-alone option"is associated with significantly lower costs and decreased invasiveness compared to lumbar instrumentation surgery, the failure of decompression in terms of SLI with need for subsequent fusion may alter the financial burden for the healthcare system significantly. On the other hand, the initial fusion without prior lumbar decompression reduces the risk of SLI, but at the same time may lead to a higher rate of adjacent segment disease (ASD) in addition to the higher invasiveness and more complications of surgery thus remaining a daily challenge. Furthermore, the definition of spinal instability often relies on low back pain, a highly subjective symptom, or radiological findings, which may not always correlate with the patient’s clinical presentation. This further complicates planning the adequate surgical treatment of patients with lumbar spinal stenosis.

In the last decade, a vast collection of clinical, radiological and technical factors, potentially influencing stability and decision-making, were identified [31, 41, 67]. Clinical findings, such as the presence of mechanical LBP, a high Body Mass Index (BMI) and previous lumbar surgery, as well as radiological factors, such as dynamic instability, disc height, facet joint angle and effusion have been discussed to account for a higher risk of SLI [40, 51, 62]. However, to date, no clear consensus in determining instability has been achieved in regard to the relative importance of these parameters. Therefore, the purpose of the present study was to review the current literature and to identify measurable preoperative radiological and clinical variables, accounting for SLI after LDS. Accordingly, a score for SLI risk prediction was generated to better identify the appropriate surgical technique in managing LSS patients with or without LDS.

## Material & methods

### Search methodology and selection criteria for detailed review

**Fig. 1:**
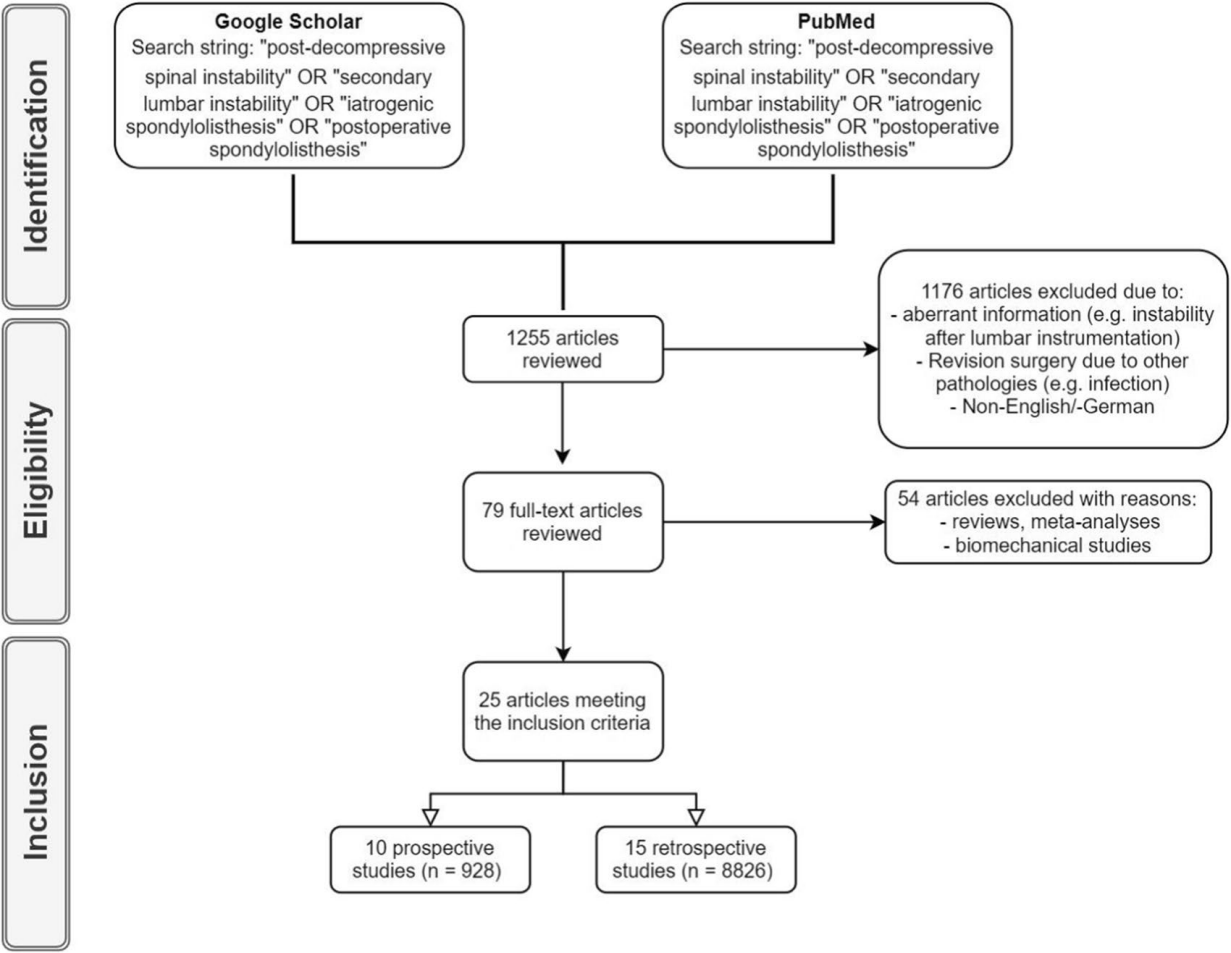
Flowchart of the study selection and literature review process

A structured narrative literature review was conducted using PubMed and Google Scholar. While not adhering to systematic review protocols (e.g., PRISMA, PROSPERO registration), we aimed to capture a broad overview of known risk factors for secondary lumbar instability. Although PubMed and Google Scholar were the primary search platforms, the included articles span key journals indexed in major databases such as MEDLINE and EMBASE. Additional references were identified through manual reference screening. The predefined search string consisted of the following keywords: “post-decompressive spinal instability”, “secondary lumbar instability”, “iatrogenic spondylolisthesis” and “postoperative spondylolisthesis”. The results of the keyword research were included for a more detailed assessment. In this study, we define"secondary lumbar instability"(SLI) as a postoperative deterioration in segmental alignment or stability following decompressive surgery for lumbar spinal stenosis, typically characterized by new or progressive olisthesis, worsening low back pain, or new neurological symptoms, and corroborated by dynamic imaging findings. During the initial screening, full-text articles in German and English language were included, if the abstracts were suitable for the literature review. Due to the limited number of articles for this specific research question, no consideration was given to the year of publication. Articles without full text availability or articles including revision surgery due to complications other than SLI were excluded. After exclusion of unsuitable literature, proof-reading of the full-text articles was performed. Additionally, the literature references of the selected articles were also screened. Reviews, meta-analyses and pre-clinical studies were excluded (Fig. 1).

### SLI scoring system

The previously identified risk factors for the development of SLI were recorded in a descriptive manner. Study type and quality as well as statistical data including possible risk factors were extracted and used for the categorization of the novel SLI risk assessment classification. Subsequently, a peer-review process within the spine surgery research group was initiated to assess the importance of individual risk factors and to assign the appropriate point weighting.

## Results

The initial database research identified 1255 papers, 1176 articles were excluded, and 79 full-text articles were reviewed, identifying 10 prospective and 15 retrospective studies suitable for the research purpose (Fig. 1). Altogether, data of 9754 patients were included and evaluated accordingly (Table 1).

**Table 1:** List of studies incorporated for the review process and development of the SLI score

Author	Year	Patient cohort	Design	Aim of the study	Findings
Lombardi et al. [45]	1985	47	Retrospective*	Facet-sparing vs. wide DS vs. fusion for degenerative listhesis	Predictor for SLI: Postoperative disc height > 6 mm
Johnsson et al. [28]	1986	45	Retrospective	Risk of SLI after DS	SLI prevalence 65% vs. 20% in patients with vs. without preop listhesis (*p* < 0.01), SLI led to a worse clinical outcome (*p* < 0.01)
Hopp et al. [20]	1988	344	Retrospective*	Evaluation of the postop development of SLI	Incidence of revision surgery due to SLI: 4.7%, women more often affected
Johnsson et al. [27]	1989	61	Retrospective	Impact of surgery extent and preop predictors for SLI	SLI occurrence men vs. women = 1:1.6 (*p* < 0.01), significant predictors: age, preop instability, surgical invasiveness, degenerative listhesis (*p* < 0.05)
Herkowitz et al. [17]	1991	50	Prospective	Evaluation of indication criteria for additional fusion in DS	Patients with DS and postop listhesis had higher postop LBP scores (2.5 vs. 1.3, *p* < 0.01) and increased need for daily pain medication
Jönsson et al. [29]	1992	60	Prospective*	Incidence of SLI in facet-joint sparing DS	SLI incidence: 2% (no preop listhesis) vs. 30% (preop listhesis)
Kotilainen et al. [39]	1993	190	Prospective	Impact of SLI on long-term HRQoL outcome	Incidence of LBP: 62% vs. 20% in patients with vs. without SLI (*p* < 0.001), ODI: 34 (w/SLI) vs. 17 (w/out SLI) (*p* = 0.001), 47% of patients w/vs. 85% of patients w/out SLI have returned to work (*p* = 0.002) at 2y FU
Schulitz et al. [63]	1995	46	Prospective	Influence of DS on SLI and impact on outcome	Hemi-facetectomy leads to increased rate of SLI, SLI incidence 30%
Fox et al. [12]	1996	124	Retrospective*	Incidence of SLI, predictors, impact on outcome in DS	SLI prevalence: 53.3%; predictors: postop physiologic disc height, higher facet joint angle (65.8° vs. 50.4°), multisegmental decompression (13% [monosegmental] vs. 59% [3-level decompression]), preop listhesis
Iguchi et al. [23]	2000	37	Retrospective	Long-term outcome of laminectomy as DS	Postop vertebral angulation of > 10° has shown with significant impact on clinical outcome (Rate of improved JOA score: 42.7 ± 31.6% (> 10°) vs. 78.5 ± 27.4% (< 10°), *p* = 0.014)
Ghogawala et al. [15]	2004	34	Prospective	Comparison of DS ± fusion in Grade I spondylolisthesis	SLI incidence in DS patients was 15%, lower postop SF-36 in DS (27.5 vs. 13.6, *p* = 0.02)
Thomé et al. [68]	2005	120	Prospective	Uni- vs. bilateral laminotomy vs. laminectomy	No significant differences regarding the SLI rate for patients treated with uni-/bilateral laminotomy or laminectomy as DS
Fu et al. [14]	2008	152	Prospective	Modified laminoforaminotomy vs. laminectomy	SLI incidence: 8% (laminectomy) vs. 0% (laminoforaminotomy), SLI was associated with increased LBP (0.05 ± 0.22 vs. 0.63 ± 1.07, *p* < 0.001)
Celik et al. [8]	2010	71	Prospective	Bilateral laminotomy vs. total laminectomy	SLI incidence in total laminectomy was 9% vs. 0% in bilateral laminotomy cohort (*p* < 0.05)
Kelleher et al. [34]	2010	75	Retrospective	Postop outcome for facet-preserving DS	Preop listhesis did not show as a significant predictor for SLI (*p* > 0.05)
Hong et al. [19]	2011	53	Retrospective	Uni- vs. bilateral DS	Significant increase of sagittal translation in patients without preop listhesis treated with bilateral DS (1.69% vs. 4.07%, *p* < 0.001), significant difference in postop sagittal translation in uni- vs. bilateral DS (2.4% vs. 4.08%, *p* = 0.047)
Lattig et al. [41]	2012	160	Retrospective	Correlation between facet joint effusion in MRI and SLI	Facet joint effusion in MRI was significantly associated with SLI (r = 0.64, *p* < 0.001)
Blumenthal et al. [5]	2013	40	Prospective	Identification of radiographic risk factors for SLI	Significant predictor: Preop motion at spondylolisthesis (OR 2.65; CI 1.08–6.46; *p* = 0.033), disc height > 6.5 mm (OR 4.1, CI 0.75–22.31), facet angle > 50°
Yang et al. [75]	2013	42	Retrospective	Risk factors for SLI	Incidence of SLI: 35.7%; Significant predictors: Asymmetrical paraspinal muscle volume, physiologic lordotic angle, facet joint tropism, smoking history
Chang et al. [9]	2014	165	Prospective	Postop outcome in patients w/or w/out preop listhesis and DS	8% overall SLI incidence at 60 months-FU, no significant SLI predictors in both cohorts, no significant differences regarding SLI
Sato et al. [59]	2015	163	Retrospective	Reoperation rate after DS, risk factors for ASD/SSD	Predictors for SLI: BMI (OR 4.13, CI 1.3–13.06, *p* = 0.02), disc height > 10 mm (OR 3.18, CI 1.03–9.82, *p* = 0.04)
Jang et al. [25]	2016	21	Retrospective	3y postop outcome in patients treated with DS for DLS	Incidence of SLI: 45%, predictor: preoperative sagittal motion (*p* < 0.01)
Ramhmdani et al. [53]	2018	105	Retrospective	Evaluation of iatrogenic spondylolisthesis	Higher preop disc height in patients with SLI at L4/5 (10.4 mm vs. 8.5 mm, *p* = 0.036)
Minamide et al. [49]	2019	218	Retrospective	Postop outcome after DS	Patients requiring subsequent fusion surgery had > 2/3 disc height loss and/or dynamic slippage of ≥ 3 mm
Urakawa et al. [69]	2020	7331	Retrospective	Rate and timing of subsequent fusion after DS and risk factor evaluation	Subsequent fusion in 6.3% vs. 14.5% of degenerative vs. isthmic spondylolisthesis at 5 years FU (*p* < 0.001); Risk factors: < 70 years (HR 1.37, CI 1.11, 1.70, *p* = 0.004), Neurogenic claudication (HR1.53, CI 1.13, 2.06, *p* = 0.006), RA/CVD (HR 1.57, CI 1.27, 1.94, *p* < 0.001)

*no statistical analysis, descriptive design; SSD same segmental disease, ASD adjacent segmental disease, DS decompressive surgery, DLS degenerative lumbar spondylolisthesis, FU follow-up, SLI secondary lumbar instability, HRQoL health-related quality of life, HR hazard ratio, RA rheumatoid arthritis, CVD collagen vascular disease, LBP low back pain, y years

### Patient-related risk factors

Patients presenting without degenerative low-grade spondylolisthesis undergoing LDS for lumbar spinal stenosis carry a 2% risk for SLI [29, 45]. In contrast, patients carrying preoperative risk factors (radiologic, surgery- or patient-related) show SLI rates of up to 70% [9, 12, 28]. Younger patients have been reported to be at higher risk for SLI after LDS [69, 75]. Recent studies revealed a roughly 40% higher risk of SLI for younger patients with a cut-off set at 70 years (hazard ratio [HR] 1.37, CI 1.11, 1.70, *p* = 0.004) [69]. Furthermore, women had a 5 times higher likelihood of being affected by postoperative slip progression than men (SLI prevalence in men vs. women: 75.0% vs. 15.8%) [20, 27, 75]. Patients presenting with postoperative slippage had a significantly increased BMI compared to patients without SLI [27, 59, 75]. A retrospective analysis of 163 patients with degenerative spondylolisthesis treated with LDS revealed that overweight patients had a fourfold higher likelihood to receive revision surgery based on SLI compared to normally weighted patients (Odd’s Ratio [OR] 4.11, confidence interval [CI] 1.29–13.11) [59]. Even the presence of rheumatoid or vascular diseases in patients with preoperative low-grade spondylolisthesis led to a significant higher risk of postoperative SLI (HR1.53, CI 1.13–2.06, *p* = 0.006) [69].

Persistent mechanical LBP as a clinical sign for facet joint affection was reported as one of the most significant predictors for postoperative instability, with cut-off values for dynamic slippage of 3 mm [14, 17, 28, 29, 34, 39, 49, 54]. Prospective study cohorts have shown an incidence of mechanical LBP of up to 62% in patients suffering from SLI due to LDS (*p* < 0.001). Moreover, LBP had a significant impact on the patient’s ability to work (return to work: 85% vs. 47%, *p* = 0.001) and the overall functional outcome (Oswestry Disability Index (ODI) 34 vs. 17 in patients with compared to patients without SLI, respectively [*p* = 0.002]) [39]. The presence of postoperative neurogenic intermittent claudication was associated with a 50% higher risk of postoperative slippage in patients with preoperative already existing degenerative or isthmic spondylolisthesis leading to fusion surgery (HR1.53, CI 1.13–2.06, *p* = 0.006) [69]. Given the prevailing ambiguity in defining LBP and its inherently subjective nature, our study endeavors to establish a more concrete and clinically applicable criterion, aiming to standardize its identification and enhance the reliability of SLI diagnoses. In this study, LBP is defined by either of the following conditions: VAS greater than 5 or the predominance of low back pain. In both scenarios, the definition additionally requires an exacerbation of symptoms upon axial loading. This dual-criterion approach aims to comprehensively encompass the varying presentations of LBP in SLI cases.

### Radiographic risk factors

Preoperative instability indicated by spondylolisthesis of 2–3 mm in dynamic lateral radiographs has been reported as a significant predictor for a postoperative deterioration resulting in SLI [5, 17, 25, 27, 29, 65, 74]. Patients with preoperative dynamic instability suffered from 2.65-fold higher risk of SLI (OR 2.65, CI 1.08–6.46, *p* = 0.033) compared to stable spondylolisthesis [5, 49]. Even the presence of a stable spondylolisthesis grade I according to Meyerding was found to be a significant predictor with a nearly threefold higher risk of SLI compared to patients without spondylolisthesis [27, 28]. A retrospective, large-cohort evaluation of long-term revision surgery rates for lumbar spinal surgical interventions revealed a roughly 10% difference in revision rates for patients treated with fusion surgery vs. LDS for degenerative spondylolisthesis (17.1% vs. 28.0%) [47]. SLI rates do not only depend on the presence of spondylolisthesis but also the type of olisthesis (degenerative vs. isthmic). Recent studies showed significant differences in subsequent revision rates at 5 year follow-up for patients with isthmic (14.5%) and degenerative spondylolisthesis (6.3%; *p* < 0.001) treated initially with LDS [69]. Further factors leading to an increased risk of postoperative instability are a sagittal facet joint angle of > 50° as well as facet joint effusion in T2-sequenced MRI as a sign of increased load bearing [5, 41]. A retrospective comparison of 124 patients treated with LDS without fusion at the level L3/4 showed significantly higher facet joint angles for those suffering from SLI (65.8° vs. 50.4°) [12]. The presence of segmental kyphosis at the index segment was associated with a significantly higher risk of a postoperative SLI deterioration (OR 0.87, CI 0.76–0.97, *p* = 0.01) [17, 23, 24]. Patients at younger age (< 70 years) and/or patients with active smoking history were at an increased risk for higher postoperative segmental kyphosis [69, 75]. A postoperatively well-preserved disc may become a risk factor for SLI, hence a retrospective analysis of predictors for SLI in 163 patients treated with LDS showed a significant impact of disc height > 6.5 mm on postoperative deterioration and revision surgery (OR 4.1, CI 0.75–22.31) [5]. This was also highlighted by another prospective study with a disc height cut-off set at 10 mm (OR 3.18, CI 1.03–9.82, *p* = 0.04) as well as several retrospective studies [12, 53, 59].

### Surgical risk factors

Multiple decompressive surgeries at the same segment accounts as a significant risk factor for postoperative SLI (OR 2.64, CI 1.13–6.17) [61]. The decompression of two or three adjacent lumbar levels resulted in a fourfold increased risk of SLI (13% in monosegmental vs. 53% and 59% in two- and three-level LDS, respectively) [12]. A comparison of uni- versus bilateral LDS showed significantly increased postoperative sagittal translation in patients without spondylolisthesis treated with bilateral LDS compared to those treated with unilateral LDS (2.4% vs. 4.1%, *p* = 0.047) [8, 14, 19].

### SLI-score

Based on the results of this review as well as the peer-review process of the authors’ spine surgery research group, the 12 most important predictors for SLI after monosegmental unilateral “over-the-top” decompression were selected and categorically divided into clinical (n = 6; mechanical LBP, age < 70 years, BMI > 30 kg/m^2^, female gender, smoking history, previous lumbar surgery [at index segment]) and radiographic risk factors (n = 6; presence of olisthesis > 5 mm, dynamic olisthesis > 3 mm, disc height > 6.5 mm, segmental kyphosis > 10°, facet joint angle > 50°, bilateral facet joint effusion > 1 mm). Mechanical LBP as well as disc height were weighted with 2 points each, as these were found to be the most important factors in both the internal peer-reviewed process and the reviewed literature. The remaining risk factors were weighted with one point each. Therefore, the SLI-score reaches a maximum of 14 points (Table 2).

**Table 2:** The SLI score

Clinical Factors	Score
Mechanical low back pain (VAS > 5 OR predominant LBP AND worsening upon axial loading)	2
Age < 70 years	1
BMI > 30 kg/m^2^	1
Female gender	1
Smoking history	1
Previous lumbar surgery (at index segment)	1
Radiologic Factors	Score
Disc height (> 6.5 mm)	2
Dynamic olisthesis (> 2 mm)	1
Presence of olisthesis (> 5 mm)	1
Segmental kyphosis (> 10°)	1
Facet joint angle (> 50°)	1
Bilateral facet joint effusion (> 1 mm)	1

## Discussion

The study aimed to identify risk factors for SLI from the published literature. Based on these results a predictive score of SLI, including the 12 most relevant risk factors reported, was created.

### Patient-related risk factors

BMI and gender are commonly reported risk factors for perioperative complications and/or inferior outcome in spinal surgery. Previous studies report a significantly higher risk to develop degenerative spinal diseases in case of obesity [13, 43, 66]. Furthermore, high body weight shows a negative impact on postoperative regeneration and patient satisfaction. Reasons include increased biomechanical stress on the spine and reduced capacity for perioperative physical activity[62], as well as chronic inflammatory processes and reduced effectiveness of pain medication. Overall, these factors lead to a reduced overall outcome with an additional higher morbidity [7, 57]. The same may account for patients presenting with a smoking history [43].

Additionally, previous studies reported on a negative impact on the overall outcome measured by the Beaujon score in case of female gender as a risk factor for SLI. This includes pain values, pain medication usage and disability [46]. Explainable reasons may include a more advanced stage of the disease at the time of surgery most likely as a result of unspecific symptoms in female patients compared to their male counterparts. This can delay in diagnosis and treatment for female patients [33]. Furthermore, postoperative pain management is known to be more challenging in females [48, 55].

Also, various previous studies reported the patient’s age may play a role in the risk for development of SLI [62]. Investigated age-cut offs may be variable and range from 65 to 75 years [1, 69]. Factors, which may be causative for the higher SLI rates in younger patients are the cascade of limited mobility, increased degeneration and vertebral auto-fusion, which tends to occur at higher age thereby decreasing the risk of a SLI [40]. The degeneration process of the lumbar spine has been categorized by Yong-Hing et al., in which the initial change of mostly discoligamentous structures (level 1) leads to an altered mobility of the spinal segment causing pain and impairment (level 2), which is then followed by a restabilization process as shown by formation of vertebral auto-fusion and osteophytic attachments (level 3) [77].

The presence of mechanical LBP was reported as a significant patient-related risk factor for SLI, yet other factors, such as concomitant osteochondrosis or ligamentous overload, should always be considered as causative factors for LBP. Spinal instability presents a fairly imprecise clinical definition, so careful clinical examination combined with adequate imaging should be used to define LBP as a potential risk factor for SLI [50].

In the complex area of secondary lumbar instability (SLI) following lumbar decompressive surgery, the significance of mechanical low back pain (LBP) as an indicator is undeniable. Despite its widespread acknowledgment in clinical practice, LBP has been challenging to define and assess precisely. This lack of a universally accepted definition has made it difficult to fully understand its influence on the development of SLI. Addressing this issue, the authors have formulated a clear and quantifiable method to evaluate LBP (VAS > 5/predominant low back pain AND exacerbation upon axial loading). Although a clear, universal definition of LBP has been elusive, it is consistently mentioned as a significant factor in the development of SLI [38, 53]. Hence, in the authors’new scoring system to assess the risk of SLI, LBP is assigned an impactful weight of 2 points out of a total of 14. Other factors in the system are also important but either do not have the same level of empirical support or are not as commonly linked to SLI as LBP. By allocating more weight to LBP, the authors highlight its crucial role in the postoperative trajectory of patients with SLI.

The last patient-related factor added to the prediction score is represented by the presence of previous lumbar surgery. Many authors report a negative influence on outcome and reoperation rates after LDS, when patients were already treated by any lumbar procedure in the past. This might be explained by an ongoing spinal disease and biomechanical alterations [62]. Accordingly, analysis showed a significant correlation between asymmetric paraspinal muscles (e.g. postoperative scar tissue) and the development of SLI postoperatively has been shown, although further studies are needed for confirmation [75]. In general, fatty degeneration of the paraspinal musculature may lead to an increased risk of postoperative slippage after LDS as a result of diminished muscular support for segmental stability replaced by scar tissue [30].

In addition, a coronal asymmetrical alignment of the intervertebral disc frequently found in patients with moderate or severe lumbar scoliosis may also play a role in the development of postoperative SLI [17]. The coronal malalignment may also be associated with asymmetrical or unbalanced activity of the paraspinal musculature prior to any operative procedure leading to a negative effect on spinal stability, as proofed in the literature [64].

Nevertheless, these factors still lacking information on exact measurement, clinical applicability and relevance and were therefore not included in the score.

### Radiographic risk factors

Whilst the clinical definition of spinal stability or instability is still based on rough estimations and scoring systems with no consensus within the scientific community, the radiographic definition is more advanced. In a landmark study conducted by White and Panjabi, spinal stability was defined as the ability of the spine to withstand displacement under physiological loading that would otherwise result in injury or irritation to the nerve roots or spinal cord. Spinal instability is thus defined as a displacement of the spine with neurological deficit, deformity, or pain [73]. Various studies aiming to show risk factors for SLI used > 3 mm in sagittal radiographic imaging as a cut-off. The most commonly reported radiological risk factors include the presence of spondylolisthesis and, in particular, dynamic olisthesis on standardized lateral x-rays. There are several proposed definitions usually based on changes in angulation or vertebral body translation. Nevertheless, there is no report of the degree of dynamic mobility constituting instability [37]. A cut-off for dynamic instability was reported at a translational movement of > 2 mm in dynamic radiographs [74].

A further factor included into our score is outlined by the presence of segmental kyphosis. This is assessed by the presence of a change in disc angulation from neutral in extension radiographs to kyphotic in flexion radiographs, accounting for segmental instability [32].

Additionally, a disc height > 6.5 mm is reported to be a significant predictor for delayed instability in the lumbar spine [5]. Presumed reasons include an auto-stabilization of segments with collapsed discs, whereas high discs bear a risk of a progressive slip over time with degeneration [5]. As disc height represents a very individual parameter, an intrinsic modification was developed, measuring disc heights in correlation to the adjacent segment and comparing the relative degree of degeneration. A measured disc height > 50% of the adjacent level was assumed to carry the risk of progressive delayed instability [40]. This fact may also support the risk factor of age < 70 years, as disc height naturally decreases with age as part of the degeneration process [75].

In consideration of the lumbar facet joints and their influence on secondary instability, facet joint angle and effusion were considered relevant factors in the literature [41]. Although the role of facet joint inclination in the development of SLI remains controversial, several studies have indicated an increased sagittal orientation as a risk factor for SLI [44, 76].

Furthermore, fluid-filled distented facet joints (> 1 mm in axial T2 weighted MRI slides) seem to predict SLI. Reasons therefore are biomechanical, as loading of the spine leads to compressive forces on the disc, resisted by the facet joints. Fluid-filled facet joints form an indirect sign for SLI as the accumulation of synovial fluid mainly occurs in patients with spinal instability. Recent studies were even able to show a direct correlation of the amount of facet joint fluid accumulation and the severity of spondylolisthesis [10]. The same accounts for synovial cysts [40, 41].

Another discussed factor in the setting of SLI seems to be presented by increased pelvic incidence (PI). Although previous studies report a positive correlation of lumbar instability and increased PI, there is no data on thresholds predicting instability [22]. Additionally, PI seems to be a very individual component, highly correlating on sagittal imbalance and compensation of the same. Therefore, it was decided not to include PI into the scoring system.

### The SLI-score

In a synopsis of all these reported risk factors, a score was assembled, aiming to predict the risk of SLI preoperatively. For the score, the 12 most commonly reported and transparent factors were merged and rated, resulting in a score of maximum 14 points. An additional requirement was constituted by the clinical usability of the score. As Kulkarni et al.[40] and Blumenthal et al. [5] have already characterized, disc height and mechanical LBP showed the most significant impact on SLI and were therefore rated with 2 points each.

## Limitations

There are several factors limiting this review. First of all, the definition of lumbar decompressive surgery is based on the authors’ standard of procedure, respectively, thereby forming a potential bias in the sense of a retrospective comparison of slightly inconsistent operative techniques with more or less impact on spinal stability. The absence of a professional librarian in the search process and the lack of a systematic review protocol (e.g., PRISMA compliance or PROSPERO registration) may limit reproducibility and comprehensiveness of our literature search. Another major factor is the inconclusive definition of spinal instability itself, which limits the assessment of the severity of each risk factor for the development of SLI. In particular, the clinical factors potentially favoring SLI are largely based on subjective data, which can be easily biased by confounders and thus also affect the validity in this review. The hereby presented score solely constitutes an overview of already described risk factors in the literature and therefore remains a theoretical basis for the concept of SLI. Furthermore, to date there is no existing data depicting the relevance of these factors, and especially on their combination. Moreover, the inclusion of clinical factors to the score may leads to less objective results due to subjective bias of clinical factors. Thus, we are not able to define clearly whether the presence of different parameters would lead to an addition or multiplication of different factors (e.g., obesity ± smoking). Besides, there are no reports on cut-off values for similar scores or expedient treatment recommendations according to when to fuse or when not to fuse, in particular depending on different surgical techniques to achieve decompression.

## Conclusion

The findings of this comprehensive review elucidate the prevailing clinical, radiographic, and patient-related risk factors, shedding light on their potential influence on postoperative outcomes in patients undergoing LDS. The introduced scoring system provides a structured framework delineating prominent risk factors for SLI, stratified based on their presumed significance in guiding clinical decision-making. While this conceptual foundation establishes a theoretical framework for understanding SLI, its practical applicability necessitates validation through rigorous clinical investigation. Nevertheless, this preliminary overview of clinically significant factors serves as a valuable resource, offering insight into characterized risk elements that can inform and potentially enhance the decision-making process in the context of LDS.

## Data Availability

No datasets were generated or analysed during the current study.

## References

[1] Aalto T, Sinikallio S, Kröger H, Viinamäki H, Herno A, Leinonen V, Turunen V, Savolainen S, Airaksinen O (2012) Preoperative predictors for good postoperative satisfaction and functional outcome in lumbar spinal stenosis surgery - A prospective observational study with a two-year follow-up. Scand J Surg 101(4):255–260 23238500 10.1177/145749691210100406

[2] Abbas J, Hamoud K, May H, Peled N, Sarig R, Stein D, Alperovitch-Najemson D, Hershkovitz I (2013) Socioeconomic and physical characteristics of individuals with degenerative lumbar spinal stenosis. Spine (Phila Pa 1976) 38(9):E554–E561 24477055 10.1097/BRS.0b013e31828a2846

[3] Abdu WA, Sacks OA, Tosteson ANA, Zhao W, Tosteson TD, Morgan TS, Pearson A, Weinstein JN, Lurie JD (2018) Long-Term results of surgery compared with nonoperative treatment for lumbar degenerative spondylolisthesis in the spine patient outcomes research trial (SPORT). Spine (Phila Pa 1976) 43(23):1619–1630 29652786 10.1097/BRS.0000000000002682 PMC6185822

[4] Atlas SJ, Keller RB, Robson D, Deyo RA, Singer DE (2000) Surgical and nonsurgical management of lumbar spinal stenosis: four-year outcomes from the maine lumbar spine study. Spine (Phila Pa 1976) 25(5):556–562 10749631 10.1097/00007632-200003010-00005

[5] Blumenthal C, Curran J, Benzel EC, Potter R, Magge SN, Harrington JF, Coumans JV, Ghogawala Z (2013) Radiographic predictors of delayed instability following decompression without fusion for degenerative Grade I lumbar spondylolisthesis: Clinical article. J Neurosurg Spine 18(4):340–346 23373567 10.3171/2013.1.SPINE12537

[6] Bridwell KH, Sedgewick TA, O’Brien MF, Lenke LG, Baldus C (1993) The role of fusion and instrumentation in the treatment of degenerative spondylolisthesis with spinal stenosis. J Spinal Disord 6(6):461–472 8130395 10.1097/00002517-199306060-00001

[7] Brill MJE, Diepstraten J, Van Rongen A, Van Kralingen S, Van Den Anker JN, Knibbe CAJ (2012) Impact of obesity on drug metabolism and elimination in adults and children. Clin Pharmacokinet. 10.2165/11599410-000000000-00000 22448619 10.2165/11599410-000000000-00000

[8] Çelik SE, Çelik S, Göksu K, Kara A, Ince I (2010) Microdecompressive laminatomy with a 5-year follow-up period for severe lumbar spinal stenosis. J Spinal Disord Tech. 10.1097/BSD.0b013e3181a3d889 20526152 10.1097/BSD.0b013e3181a3d889

[9] Chang HS, Fujisawa N, Tsuchiya T, Oya S, Matsui T (2014) Degenerative spondylolisthesis does not affect the outcome of unilateral laminotomy with bilateral decompression in patients with lumbar stenosis. Spine (Phila Pa 1976) 39(5):400–408 24365897 10.1097/BRS.0000000000000161

[10] Cho BY, Murovic JA, Park J (2009) Imaging correlation of the degree of degenerative L4–5 spondylolisthesis with the corresponding amount of facet fluid: Clinical article. J Neurosurg Spine. 10.3171/2009.6.SPINE08413 19929367 10.3171/2009.6.SPINE08413

[11] Deyo RA, Mirza SK, Martin BI, Kreuter W, Goodman DC, Jarvik JG (2010) Trends, major medical complications, and charges associated with surgery for lumbar spinal stenosis in older adults. JAMA 303(13):1259–1265 20371784 10.1001/jama.2010.338 PMC2885954

[12] Fox MW, Onofrio BM, Hanssen AD (1996) Clinical outcomes and radiological instability following decompressive lumbar laminectomy for degenerative spinal stenosis: A comparison of patients undergoing concomitant arthrodesis versus decompression alone. J Neurosurg 85(5):793–802 8893716 10.3171/jns.1996.85.5.0793

[13] Fransen M, Woodward M, Norton R, Coggan C, Dawe M, Sheridan N (2002) Risk factors associated with the transition from acute to chronic occupational back pain. Spine (Phila Pa 1976). 10.1097/00007632-200201010-00022 11805644 10.1097/00007632-200201010-00022

[14] Fu YS, Zeng BF, Xu JG (2008) Long-term outcomes of two different decompressive techniques for lumbar spinal stenosis. Spine (Phila Pa 1976) 33(5):514–518 18317196 10.1097/BRS.0b013e3181657dde

[15] Ghogawala Z, Benzel EC, Amin-Hanjani S, Barker FG, Harrington JF, Magge SN, Strugar J, Coumans JVCE, Borges LF (2004) Prospective outcomes evaluation after decompression with or without instrumented fusion for lumbar stenosis and degenerative Grade I spondylolisthesis. J Neurosurg Spine 1(3):267–272 15478364 10.3171/spi.2004.1.3.0267

[16] Golinvaux NS, Basques BA, Bohl DD, Yacob A, Grauer JN (2015) Comparison of 368 Patients Undergoing Surgery for Lumbar Degenerative Spondylolisthesis from the SPORT Trial with 955 from the NSQIP Database. Spine (Phila Pa 1976) 40(5):342–348 25757036 10.1097/BRS.0000000000000747

[17] Herkowitz HN, Kurz LT (1991) Degenerative lumbar spondylolisthesis with spinal stenosis. A prospective study comparing decompression with decompression and intertransverse process arthrodesis. J Bone Joint Surg Am 73(6):802–808 2071615

[18] Hermansen E, Indrekvam K, Franssen E et al (2024) A randomized trial on three different minimally invasive decompression techniques for lumbar spinal stenosis. Five years follow-up from the NORDSTEN-SST. Eur Spine J 5:1–10 10.1007/s00586-024-08514-0 39448401

[19] Hong SW, Choi KY, Ahn Y, Baek OK, Wang JC, Lee SH, Lee HY (2011) A comparison of unilateral and bilateral laminotomies for decompression of L4-L5 spinal stenosis. Spine (Phila Pa 1976). 10.1097/BRS.0b013e3181db998c 21192307 10.1097/BRS.0b013e3181db998c

[20] Hopp E, Tsou PM (1988) Postdecompression lumbar instability. Clin Orthop Relat Res 227:143–151 2962798

[21] Horan J, Ben HM, Bolger C (2021) Bilateral laminotomy through a unilateral approach (minimally invasive) versus open laminectomy for lumbar spinal stenosis. Br J Neurosurg 35(2):161–165 32530321 10.1080/02688697.2020.1777253

[22] Hsieh MK, Kao FC, Chen WJ, Chen IJ, Wang SF (2018) The influence of spinopelvic parameters on adjacent-segment degeneration after short spinal fusion for degenerative spondylolisthesis. J Neurosurg Spine. 10.3171/2018.2.SPINE171160 30028254 10.3171/2018.2.SPINE171160

[23] Iguchi T, Kurihara A, Nakayama J, Sato K, Kurosaka M, Yamasaki K (2000) Minimum 10-year outcome of decompressive laminectomy for degenerative lumbar spinal stenosis. Spine (Phila Pa 1976). 10.1097/00007632-200007150-00003 10888941 10.1097/00007632-200007150-00003

[24] Inose H, Kato T, Onuma H, Morishita S, Matsukura Y, Yuasa M, Hirai T, Yoshii T, Okawa A (2021) Predictive factors affecting surgical outcomes in patients with degenerative lumbar spondylolisthesis. Spine (Phila Pa 1976). 10.1097/BRS.0000000000003944 33428364 10.1097/BRS.0000000000003944

[25] Jang JW, Park JH, Hyun SJ, Rhim SC (2016) Clinical outcomes and radiologic changes after microsurgical bilateral decompression by a unilateral approach in patients with lumbar spinal stenosis and grade I degenerative spondylolisthesis with a minimum 3-year follow-Up. Clin Spine Surg 29(7):268–271 23073148 10.1097/BSD.0b013e31827566a8

[26] Jarrett MS, Orlando JF, Grimmer-Somers K (2012) The effectiveness of land based exercise compared to decompressive surgery in the management of lumbar spinal-canal stenosis: a systematic review. BMC Musculoskelet Disord 13:30 22369653 10.1186/1471-2474-13-30 PMC3305601

[27] Johnsson KE, Redlund-Johnell I, Udén A, Willner S (1989) Preoperative and postoperative instability in lumbar spinal stenosis. Spine (Phila Pa 1976) 14(6):591–593 2749373 10.1097/00007632-198906000-00008

[28] Johnsson KE, Willner S, Johnsson K (1986) Postoperative instability after decompression for lumbar spinal stenosis. Spine (Phila Pa 1976) 11(2):107–110 3704799 10.1097/00007632-198603000-00001

[29] Jönsson B, Åkesson M, Jonsson K, Strömqvist B (1992) Low risk for vertebral slipping after decompression with facet joint preserving technique for lumbar spinal stenosis. Eur Spine J 1(2):100–104 20054955 10.1007/BF00300935

[30] Kalichman L, Hodges P, Li L, Guermazi A, Hunter DJ (2010) Changes in paraspinal muscles and their association with low back pain and spinal degeneration: CT study. Eur Spine J 19(7):1136–1144 20033739 10.1007/s00586-009-1257-5 PMC2900015

[31] Kalichman L, Suri P, Guermazi A, Li L, Hunter DJ (2009) Facet orientation and tropism: associations with facet joint osteoarthritis and degeneratives. Spine (Phila Pa 1976) 34(16):E579–E585 19770601 10.1097/BRS.0b013e3181aa2acb PMC3892429

[32] Kanayama M, Hashimoto T, Shigenobu K, Oha F, Ishida T, Yamane S (2003) Intraoperative biomechanical assessment of lumbar spinal instability: validation of radiographic parameters indicating anterior column support in lumbar spinal fusion. Spine (Phila Pa 1976). 10.1097/01.BRS.0000085357.24025.27 10.1097/01.BRS.0000085357.24025.27 14560085

[33] Katz JN, Wright EA, Guadagnoli E, Liang MH, Karlson EW, Cleary PD (1994) Differences between men and women undergoing major orthopedic surgery for degenerative arthritis. Arthritis Rheum. 10.1002/art.1780370512 8185695 10.1002/art.1780370512

[34] Kelleher MO, Timlin M, Persaud O, Rampersaud YR (2010) Success and failure of minimally invasive decompression for focal lumbar spinal stenosis in patients with and without deformity. Spine (Phila Pa 1976). 10.1097/BRS.0b013e3181c46fb4 10.1097/BRS.0b013e3181c46fb4 20386501

[35] Kepler CK, Vaccaro AR, Hilibrand AS, Anderson DG, Rihn JA, Albert TJ, Radcliff KE (2014) National trends in the use of fusion techniques to treat degenerative spondylolisthesis. Spine (Phila Pa 1976) 39(19):1584–1589 24979276 10.1097/BRS.0000000000000486

[36] Kgomotso EL, Hellum C, Fagerland MW et al (2024) Decompression alone or with fusion for degenerative lumbar spondylolisthesis (Nordsten-DS): five year follow-up of a randomised, multicentre, non-inferiority trial. BMJ 386:e079771 39111800 10.1136/bmj-2024-079771 PMC11304163

[37] Kim HS, Il JuC, Kim SW, Kang JH (2015) Lying down instability undetected on standing dynamic radiographs. J Korean Neurosurg Soc. 10.3340/jkns.2015.58.6.560 26819694 10.3340/jkns.2015.58.6.560 PMC4728097

[38] Kleinstueck FS, Fekete TF, Mannion AF, Grob D, Porchet F, Mutter U, Jeszenszky D (2012) To fuse or not to fuse in lumbar degenerative spondylolisthesis: Do baseline symptoms help provide the answer? Eur Spine J 21(2):268–275 21786174 10.1007/s00586-011-1896-1 PMC3265591

[39] Kotilainen E, Valtonen S (1993) Clinical instability of the lumbar spine after microdiscectomy. Acta Neurochir (Wien) 125(1–4):120–126 8122535 10.1007/BF01401838

[40] Kulkarni AG, Kunder TS, Dutta S (2020) Degenerative spondylolisthesis: when to fuse and when not to? A new scoring system. Clin Spine Surg 33(8):E391–E400 32187081 10.1097/BSD.0000000000000970

[41] Lattig F, Fekete TF, Grob D, Kleinstück FS, Jeszenszky D, Mannion AF (2012) Lumbar facet joint effusion in MRI: a sign of instability in degenerative spondylolisthesis? Eur Spine J Off Publ Eur Spine Soc Eur Spinal Deform Soc Eur Sect Cerv Spine Res Soc 21(2):276–281 10.1007/s00586-011-1993-1 PMC3265597 21932065

[42] Lener S, Schmölz W, Abramovic A, Kluger P, Thomé C, Hartmann S (2023) The effect of various options for decompression of degenerated lumbar spine motion segments on the range of motion: a biomechanical in vitro study. Eur Spine J 32(4):1358–1366 36826599 10.1007/s00586-023-07587-7

[43] Lener S, Wipplinger C, Hartmann S, Thomé C, Tschugg A (2020) The impact of obesity and smoking on young individuals suffering from lumbar disc herniation: a retrospective analysis of 97 cases. Neurosurg Rev. 10.1007/s10143-019-01151-y 31414196 10.1007/s10143-019-01151-y PMC7515935

[44] Liu Z, Duan Y, Rong X, Wang B, Chen H, Liu H (2017) Variation of facet joint orientation and tropism in lumbar degenerative spondylolisthesis and disc herniation at L4–L5: A systematic review and meta-analysis. Clin Neurol Neurosurg. 10.1016/j.clineuro.2017.08.005 28843706 10.1016/j.clineuro.2017.08.005

[45] Lombardi JS, Wiltse LL, Reynolds J, Widell EH, Spencer C (1985) Treatment of degenerative spondylolisthesis. Spine (Phila Pa 1976) 10(9):821–827 4089657 10.1097/00007632-198511000-00008

[46] Mariconda M, Zanforlino G, Celestino GA, Brancaleone S, Fava R, Milano C (2000) Factors influencing the outcome of degenerative lumbar spinal stenosis. J Spinal Disord. 10.1097/00002517-200004000-00007 10780688 10.1097/00002517-200004000-00007

[47] Martin BI, Mirza SK, Comstock BA, Gray DT, Kreuter W, Deyo RA (2007) Reoperation rates following lumbar spine surgery and the influence of spinal fusion procedures. Spine (Phila Pa 1976) 32(3):382–387 17268274 10.1097/01.brs.0000254104.55716.46

[48] Meibohm B, Beierle I, Derendorf H (2002) How important are gender differences in pharmacokinetics? Clin Pharmacokinet. 10.2165/00003088-200241050-00002 12036391 10.2165/00003088-200241050-00002

[49] Minamide A, Simpson AK, Okada M et al (2019) Microendoscopic decompression for lumbar spinal stenosis with degenerative spondylolisthesis: the influence of spondylolisthesis stage (Disc Height and Static and Dynamic Translation) on clinical outcomes. Clin Spine Surg 32(1):E20–E26 30222618 10.1097/BSD.0000000000000710

[50] Pao J-L, Chen W-C, Chen P-Q (2009) Clinical outcomes of microendoscopic decompressive laminotomy for degenerative lumbar spinal stenosis. Eur Spine J 18(5):672–678 19238459 10.1007/s00586-009-0903-2 PMC3234002

[51] Papavero L, Thiel M, Fritzsche E, Kunze C, Westphal M, Kothe R (2009) Lumbar spinal stenosis: prognostic factors for bilateral microsurgical decompression using a unilateral approach. Neurosurgery 65(6 Suppl):182–187 (discussion187) 19934993 10.1227/01.NEU.0000341906.65696.08

[52] Pietrantonio A, Trungu S, Famà I, Forcato S, Miscusi M, Raco A (2019) Long-term clinical outcomes after bilateral laminotomy or total laminectomy for lumbar spinal stenosis: a single-institution experience. Neurosurg Focus 46(5):E2 31042648 10.3171/2019.2.FOCUS18651

[53] Ramhmdani S, Xia Y, Xu R, Kosztowski T, Sciubba D, Witham T, Bydon A (2018) Iatrogenic spondylolisthesis following open lumbar laminectomy: case series and review of the literature. World Neurosurg 113:e383–e390 29454126 10.1016/j.wneu.2018.02.039

[54] Rampersaud YR, Fisher C, Yee A, Dvorak MF, Finkelstein J, Wai E, Abraham E, Lewis SJ, Alexander D, Oxner W (2014) Health-related quality of life following decompression compared to decompression and fusion for degenerative lumbar spondylolisthesis: a Canadian multicentre study. Can J Surg. 10.1503/cjs.032213 25078938 10.1503/cjs.032213 PMC4119126

[55] Rhudy JL, Bartley EJ (2010) The effect of the menstrual cycle on affective modulation of pain and nociception in healthy women. Pain. 10.1016/j.pain.2010.02.041 20304557 10.1016/j.pain.2010.02.041

[56] Rihn JA, Hilibrand AS, Zhao W, Lurie JD, Vaccaro AR, Albert TJ, Weinstein J (2015) Effectiveness of surgery for lumbar stenosis and degenerative spondylolisthesis in the octogenarian population: analysis of the Spine Patient Outcomes Research Trial (SPORT) data. J Bone Jt Surg - Am 97(3):177–185 10.2106/JBJS.N.00313 PMC4310864 25653317

[57] Risbud MV, Shapiro IM (2014) Role of cytokines in intervertebral disc degeneration: pain and disc content. Nat Rev Rheumatol. 10.1038/nrrheum.2013.160 24166242 10.1038/nrrheum.2013.160 PMC4151534

[58] Rosenberg NJ (1976) Degenerative spondylolisthesis: surgical treatment. Clin Orthop Relat Res 117:112–120 132322

[59] Sato S, Yagi M, Machida M et al (2015) Reoperation rate and risk factors of elective spinal surgery for degenerative spondylolisthesis: minimum 5-year follow-up. Spine J 15(7):1536–1544 25681581 10.1016/j.spinee.2015.02.009

[60] Schizas C, Schmit A, Schizas A, Becce F, Kulik G, Pierzchała K (2014) Secular changes of spinal canal dimensions in Western Switzerland: a narrowing epidemic? Spine (Phila Pa 1976) 39(17):1339–1344 24875965 10.1097/BRS.0000000000000445

[61] Schöller K, Alimi M, Cong G-T, Christos P, Navarro-Ramirez R, Härtl R (2016) Lumbar spinal stenosis associated with degenerative lumbar spondylolisthesis: a systematic review and meta-analysis of secondary fusion rates following open vs. minimally invasive decompression. Glob Spine J 6(1_suppl):s-0036–1582973-s-0036–1582973 10.1093/neuros/nyw091 28362963

[62] Schöller K, Steingrüber T, Stein M, Vogt N, Müller T, Pons-Kühnemann J, Uhl E (2016) Microsurgical unilateral laminotomy for decompression of lumbar spinal stenosis: long-term results and predictive factors. Acta Neurochir (Wien) 158(6):1103–1113 27084380 10.1007/s00701-016-2804-6

[63] Schulitz KP (1995) Risk of instability following decompression surgery in lumbar stenosis. Z Orthop Ihre Grenzgeb 133(3):236–241 7610705 10.1055/s-2008-1039443

[64] Shafaq N, Suzuki A, Matsumura A, Terai H, Toyoda H, Yasuda H, Ibrahim M, Nakamura H (2012) Asymmetric degeneration of paravertebral muscles in patients with degenerative lumbar scoliosis. Spine (Phila Pa 1976) 37(16):1398–1406 22322373 10.1097/BRS.0b013e31824c767e

[65] Sheehan JM, Shaffrey CI, Jane JA Sr (2001) Degenerative lumbar stenosis: the neurosurgical perspective - PubMed. Clin Orthop Relat Res 384:61–74 11249181

[66] Sheng B, Feng C, Zhang D, Spitler H, Shi L (2017) Associations between obesity and spinal diseases: a medical expenditure panel study analysis. Int J Environ Res Public Health. 10.3390/ijerph14020183 28208824 10.3390/ijerph14020183 PMC5334737

[67] Simmonds AM, Rampersaud YR, Dvorak MF, Dea N, Melnyk AD, Fisher CG (2015) Defining the inherent stability of degenerative spondylolisthesis: a systematic review. J Neurosurg Spine 23(2):178–189 25978079 10.3171/2014.11.SPINE1426

[68] Thomé C, Zevgaridis D, Leheta O, Bäzner H, Pöckler-Schöniger C, Wöhrle J, Schmiedek P (2005) Outcome after less-invasive decompression of lumbar spinal stenosis: a randomized comparison of unilateral laminotomy, bilateral laminotomy, and laminectomy. J Neurosurg Spine 3(2):129–141 16370302 10.3171/spi.2005.3.2.0129

[69] Urakawa H, Jones T, Samuel A et al (2020) The necessity and risk factors of subsequent fusion after decompression alone for lumbar spinal stenosis with lumbar spondylolisthesis: 5 years follow-up in two different large populations. Spine J 20(10):1566–1572 32417500 10.1016/j.spinee.2020.04.026

[70] Weinstein JN, Lurie JD, Tosteson TD et al (2009) Surgical compared with nonoperative treatment for lumbar degenerative spondylolisthesis: four-year results in the Spine Patient Outcomes Research Trial (SPORT) randomized and observational cohorts. J Bone Jt Surg 91(6):1295–1304 10.2106/JBJS.H.00913 PMC2686131 19487505

[71] Weinstein JN, Tosteson TD, Lurie JD et al (2008) Surgical versus nonsurgical therapy for lumbar spinal stenosis. N Engl J Med 358(8):794–810 18287602 10.1056/NEJMoa0707136 PMC2576513

[72] Weinstein JN, Tosteson TD, Lurie JD, Tosteson ANA, Hanscom B, Skinner JS, Abdu WA, Hilibrand AS, Boden SD, Deyo RA (2006) Surgical vs nonoperative treatment for lumbar disk herniation. The Spine Patient Outcomes Research Trial (SPORT): a randomized trial. JAMA. 10.1001/jama.296.20.2441 10.1001/jama.296.20.2441 PMC2553805 17119140

[73] White AA, Johnson RM, Panjabi MM, Southwick WO (1975) Biomechanical analysis of clinical stability in the cervical spine. CLINORTHOP No 109(109):85–96 10.1097/00003086-197506000-00011 1132209

[74] Wiltse LL, Winter RB (1983) Terminology and measurement of spondylolisthesis. J Bone Jt Surg - Ser A. 10.2106/00004623-198365060-00007 6863359

[75] Yang JC, Kim SG, Kim TW, Park KH (2013) Analysis of factors contributing to postoperative spinal instability after lumbar decompression for spinal stenosis. Korean J Spine 10(3):149 24757477 10.14245/kjs.2013.10.3.149 PMC3941765

[76] Yao Q, Wang S, Shin JH, Li G, Wood KB (2013) Lumbar facet joint motion in patients with degenerative spondylolisthesis. J Spinal Disord Tech. 10.1097/BSD.0b013e31827a254f 23168388 10.1097/BSD.0b013e31827a254f PMC3554875

[77] Yong Hing K, Kirkaldy Willis WH (1983) The pathophysiology of degenerative disease of the lumbar spine. Orthop Clin North Am. 10.1016/s0030-5898(20)31329-8 6346204

